# Reduced-intensity conditioning is effective for allogeneic hematopoietic stem cell transplantation in infants with *MECOM*-associated syndrome

**DOI:** 10.1007/s12185-022-03505-7

**Published:** 2022-12-14

**Authors:** Masahiro Irie, Tetsuya Niihori, Tomohiro Nakano, Tasuku Suzuki, Saori Katayama, Kunihiko Moriya, Hidetaka Niizuma, Nobu Suzuki, Yuka Saito-Nanjo, Masaei Onuma, Takeshi Rikiishi, Atsushi Sato, Mayumi Hangai, Mitsuteru Hiwatari, Junji Ikeda, Reo Tanoshima, Norio Shiba, Yuki Yuza, Nobuyuki Yamamoto, Yoshiko Hashii, Motohiro Kato, Junko Takita, Miho Maeda, Yoko Aoki, Masue Imaizumi, Yoji Sasahara

**Affiliations:** 1 Department of Pediatrics, Tohoku University Graduate School of Medicine, 1-1 Seiryo-Machi, Aoba-Ku, Sendai, Miyagi 980-8574 Japan; 2 Department of Medical Genetics, Tohoku University Graduate School of Medicine, Miyagi, Japan; 3 Department of Hematology and Oncology, Miyagi Children’s Hospital, Miyagi, Japan; 4 Department of Pediatrics, The University of Tokyo, Tokyo, Japan; 5 Department of Pediatrics, School of Medicine, Teikyo University, Tokyo, Japan; 6 Department of Pediatrics, Yokohama City University Graduate School of Medicine, Kanagawa, Japan; 7 Department of Hematology and Oncology, Tokyo Metropolitan Children’s Medical Center, Tokyo, Japan; 8 Department of Pediatrics, Kobe University Graduate School of Medicine, Hyogo, Japan; 9 Department of Pediatrics, Osaka University Graduate School of Medicine, Osaka, Japan; 10 Department of Pediatrics, Osaka International Cancer Institute, Osaka, Japan; 11 Children’s Cancer Center, National Center for Child Health and Development, Tokyo, Japan; 12 Department of Pediatrics, Kyoto University Graduate School of Medicine, Kyoto, Japan; 13 Department of Pediatrics, Nippon Medical School, Tokyo, Japan

**Keywords:** Inherited bone marrow failure syndrome, Radio-ulnar synostosis with amegakaryocytic thrombocytopenia, *MECOM*-associated syndrome, Reduced-intensity conditioning, Allogeneic hematopoietic stem cell transplantation

## Abstract

Mutations in the *MECOM* encoding EVI1 are observed in infants who have radioulnar synostosis with amegakaryocytic thrombocytopenia. *MECOM*-associated syndrome was proposed based on clinical heterogeneity. Allogeneic hematopoietic stem cell transplantation (HSCT) is a curative treatment for progressive bone marrow failure. However, data regarding allogeneic HSCT for this rare disease are limited. We retrospectively assessed overall survival, conditioning regimen, regimen-related toxicities and long-term sequelae in six patients treated with allogeneic HSCT. All patients received a reduced-intensity conditioning (RIC) regimen consisting of fludarabine, cyclophosphamide or melphalan, and rabbit anti-thymocyte globulin and/or low-dose total body/thoracic-abdominal/total lymphoid irradiation, followed by allogeneic bone marrow or cord blood transplantation from unrelated donors between 4 and 18 months of age. All patients survived and achieved stable engraftment and complete chimerization with the donor type. Moreover, no patient experienced severe regimen-related toxicities, and only lower grades of acute graft-versus-host disease were observed. Three patients treated with low-dose irradiation had relatively short stature compared to three patients not treated with irradiation. Therefore, allogeneic HSCT with RIC is an effective and feasible treatment for infants with *MECOM*-associated syndrome. Future studies are needed to evaluate the use of low-dose irradiation to avoid risks of other long-term sequelae.

## Introduction

Radioulnar synostosis with amegakaryocytic thrombocytopenia (RUSAT) is an inherited bone marrow failure syndrome (IBMFS). This condition is characterized by thrombocytopenia, which progresses to pancytopenia, and congenital proximal fusion of the radius and ulna [1]. In a previous report, two unrelated families presented with RUSAT caused by *HOXA11* mutations [2]. However, not all cases of RUSAT are due to *HOXA11* mutations, and additional genetic loci are also responsible for this condition [3].

We initially reported three patients with RUSAT who presented with heterozygous missense mutations in the *MECOM* encoding the oncoprotein EVI1. These missense mutations were clustered within the 8th zinc finger motif, localized at the C-terminus of the *MECOM*. Moreover, functional assays revealed the critical role of EVI1 in normal hematopoiesis and the development of forelimbs and fingers in humans [4].

The *MECOM*-associated syndrome, a recently discovered disease, was proposed based on clinical findings. That is, patients with *MECOM* mutations have clinical phenotypic heterogeneity for BMF and proximal radioulnar synostosis (RUS). A previous study presented 12 patients, including familial and sporadic patients, with germline mutation in the *MECOM*, and their broad clinical spectrum ranged from isolated RUS with or without mild hematological abnormalities to severe IBMFS without evident skeletal abnormalities [5].

Allogeneic hematopoietic stem cell transplantation (HSCT) is a curative treatment for progressive bone marrow failure in patients with *MECOM*-associated syndrome. However, the appropriate conditioning regimens for this condition is yet to be determined, and the characteristics of early infants treated with HSCT have not been evaluated.

Hence, in this study, we analyzed the clinical outcomes of six patients treated with reduced-intensity conditioning (RIC) regimens and allogeneic HSCT. We provided insights on the effectivity of these regimens as well as their associated risks for infants with *MECOM*-associated syndrome.

## Patients and methods

WE retrospectively summarized the clinical and genetic profiles of six patients with *MECOM*-associated syndrome who were treated with allogeneic HSCT and reported in literatures or abstracts in Japan. These included family history, sex, weeks of gestation, initial clinical findings, presence of bone and other abnormalities, hematological data, transfusion dependency, age at progression to pancytopenia, type of *MECOM* mutation, and alterations in the EVI1 protein.

Moreover, we summarized the data on overall survival rate, age at transplantation, source of HSC, human leukocyte antigen (HLA) compatibility, type of conditioning regimen, number of total infused nuclear cells, status of bone marrow chimera, administration of graft-versus-host disease (GVHD) prophylaxis, grades of acute GVHD, chronic GVHD, presence of regimen-related toxicities, and long-term sequelae.

Statistical analysis was performed using Student’s *t* test, and a *p* value < 0.01 was considered statistically significant.

This study was approved by ethics committee of Tohoku University Graduate School of Medicine, and written informed consent was obtained from the patients’ parents.

## Results

### Patients’ characteristics

The clinical and genetic profiles of the six patients (Pts) with *MECOM*-associated syndrome enrolled in this study are shown in Table 1. Pts 5 and 6 had a family history of RUS, and with Pt 6 having maternal history of mild hematological abnormality. Meanwhile, four patients had de novo mutations in *MECOM*. Initial findings included petechiae, pulmonary bleeding, severe anemia, and fatal distress. Pts 1–3 presented with RUS and bone abnormality. Meanwhile, Pts 4–6 had no RUS and Pt 5 had bone abnormality at birth. Pts 2 and 3 had hearing disability. All patients rapidly progressed to severe pancytopenia or bicytopenia between 0 and 5 months of age, and all of them required repeated transfusion to prevent severe bleeding and anemia. Moreover, they were at a high risk of life-threatening infections due to severe neutropenia. Thus, prophylactic antibiotics and antifungal agents were required.

**Table 1:** Clinical and genetic profiles of six patients with *MECOM*-associated syndrome in this study

Pt No	1	2	3	4	5	6
Family history	no	no	no	no	RUS in father and uncle Clinodactyly in brother, father, and uncle	RUS, congenital left clubfoot, bilateral CDH, chronic thrombocytopenia and transient leukopenia in mother
Weeks of gestation (weeks)	35	37	31	40	37	38
Birth weight (g)	2160	2058	2180	2936	2368	3414
Gender	Female	Female	Male	Female	Female	Female
Initial findings	Fetal distress Systemic Petechiae Severe anemia	Systemic petechiae	Fetal hydrops Severe anemia	Pulmonary bleeding Severe anemia Thrombocytopenia	Systemic petechiae Severe anemia	Systemic petechiae Severe anemia ICH, Convulsion
Bone abnormalities	RUS Bilateral bony defect of the intermediate phalanges of the fifth digits	RUS Bilateral clinodactyly of the fifth digits	RUS Overlapping fingers	No	Bilateral clinodactyly of the fifth digits	No
Hearing	Normal	Sensorineural hearing impairment: Rt 55 dB, Lt 34 dB	Prelingual sensorineural hearing impairment: Rt 60 dB, Lt 25 dB	Normal	Normal	Normal
Leukocyte count at birth (/mm^3^)	6780	17,100	3220	14,220	10,700	7600
Hemoglobin count at birth (g/dL)	4.0	12.9	2.7	7.2	7.3	6.3
Platelet count at birth (/mm^3^)	5000	8000	89,000	9000	4000	7000
Transfusion dependency	RBC, PC	RBC, PC	RBC, PC	RBC, PC	RBC, PC	RBC, PC⇒PC only
Progression to pancytopenia (months)	2	5	at birth	2	4	Bicytopenia only
Heterozygous mutations of MECOM gene	c.2266A > G	c.2252A > G	c.2248C > T	c.2248C > T, somatic mosaicism	c.2208-4A > G	c.2285 + 1 G > A, LOH
Alteration of EVI1 protein	p.Thr756Ala	p.His751Arg	p.Arg750Trp	p.Arg750Trp	p.Cys735-Arg736 ins Ser	exon 11 skipping and ins intron 11

*Pt* patient, *RUS* radioulnar synostosis, *CDH* congenital dislocation of the hip joint, *ICH* intracranial hemorrhage, *Rt* right, *Lt* left, *RBC* red blood cell concentrate, *PC* platelet concentrate *LOH* loss of heterozygosity

**Fig. 1:**
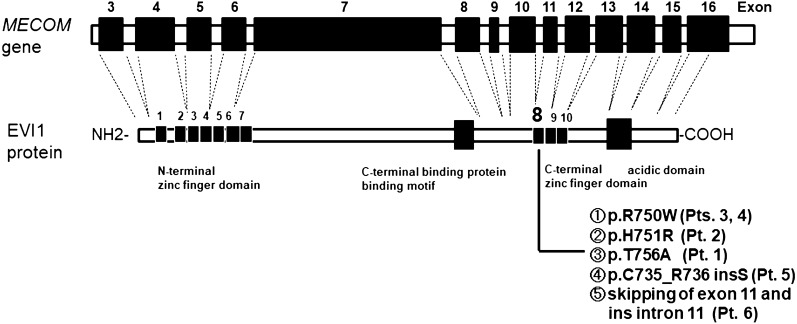
Summary of *MECOM* mutations in six patients with *MECOM*-associated syndrome enrolled in this study [4, 6, 7]

All heterozygous missense and splice-site mutations were clustered within the 8th zinc finger motif, localized at the C-terminus of the *MECOM*, as described previously (Fig. 1). Among the four patients with de novo mutations in *MECOM* gene, Pts 1–3 had heterozygous de novo missense mutations (c.2248C > T [p.Arg750Trp], c.2252A > G [p.His751Arg], and c.2266A > G [p.Thr756Ala]) [4], while Pt 4 had a heterozygous de novo mutation (c.2248C > T [p.Arg750Trp]) and somatic mosaicism in the *MECOM* [6]. Pt 5 and her brother, father, and uncle had heterozygous splice-site mutations (c.2208-4A > G), resulting in p.Cys735_Arg736insSer (CAG insertion) of the EVI1 protein. Further, Pt 6 and her mother had a heterozygous splice-site mutation (c.2285 + 1G > A), resulting in skipping of exon 11 including the 8th zinc finger motif and insertion of intron 11, and somatic loss of heterozygosity (LOH) which reduced the allele fraction of the mutation in blood cells [7].

### Donor, stem cell source and GVHD prophylaxis

Data on RIC and allogeneic HSCT are shown in Table 2. The patient’s age at HSCT was between 4 and 18 months. The sources of donor cells were bone marrow from an unrelated donor in three patients and unrelated cord blood in the other three patients. The number of total infused nuclear cells was sufficient for engraftment in all patients. HLA compatibility was 8/8 or 7/8 matched in alleles in unrelated bone marrow transplantations and 7/8 or 4/8 matched in alleles in cord blood transplantation (CBT). Regarding GVHD prophylaxis, five patients received tacrolimus (FK506) and short-term methotrexate (MTX) while one patient received cyclosporin A (CyA) and short-term MTX.

**Table 2:** Summary of six patients treated with RIC and allogeneic HSCT

Pt. No (age at transplant)	Donor source	Conditioning regimen	Infused total cell counts (/kg)	HLA compatibility	GVHD prophylaxis	Neutrophils > 500/mm^3^	Platelets > 50,000/mm^3^	Chimerism	Regimen-related toxicity	aGVHD	cGVHD	Age at last follow up (y)	Body height at last follow up [cm (SD)]	Body weight at last follow up [kg (SD)]	Other
1 (4 months)	Unrelated CB	FLU 0.83 mg/kg × 5 L-PAM 2.3 mg/kg × 2 rATG 1.25 mg/kg × 1	21.2 × 10^7^	Allele 7/8 match DR 1 locus mismatch	Oral CyA short-term MTX	Day + 14	Day + 22	BM complete chimera at day + 35	Mucositis grade 1	Skin stage 3 (grade 2)	No	8.0	116.0 (− 1.61)	17.0 (− 2.85)	
2 (18 months)	Unrelated BM	FLU 25 mg/m2 × 4 CY 50 mg/kg × 4 rATG 2.5 mg/kg × 4 TLI 3 Gy	6.9 × 10^8^	Allele 8/8 match	FK506 Short-term MTX	Day + 16	Day + 27	BM complete chimera at day + 60	Generalized convulsion at day 1 MRSA sepsis at day 60	Skin stage 3 (grade 2)	No	14.1	139.5 (− 3.01)	32.3 (− 3.15)	
3 (8 months)	Unrelated BM	FLU 0.83 mg/kg × 5 CY 50 mg/kg × 4 TAI 2 Gy	2.7 × 10^8^	Allele 7/8 match DR 1 locus mismatch	FK506 short-term MTX	Day + 6	Day + 23	BM complete chimera at day + 23	no	Skin stage 1 (grade 1)	No	11.7	134.8 (− 1.61)	28.0 (− 1.85)	GH replacement therapy ( +)
4 (5 months)	Unrelated CB	FLU 1 mg/kg × 5 L-PAM 2.3 mg/kg × 2 TBI 3 Gy	12.7 × 10^7^	Allele 7/8 match DR 1 locus mismatch	FK506 short-term MTX	Day + 18	Day + 35	BM complete chimera at day + 36	VOD grade 1	Skin stage 2 (grade 1)	No	4.2	87.8 (− 3.08)	11.6 (− 1.82)	
5 (8 months)	Unrelated CB	FLU 0.83 mg/kg × 5 L-PAM 2.3 mg/kg × 2 rATG 1.25 mg/kg × 1	17.2 × 10^7^	Allele 4/8 match DR 2 loci, C 2 loci mismatch	FK506 short-term MTX	Day + 22	Day + 34	BM complete chimera at day + 30	no	Skin stage 1 (grade 1)	No	3.7	97.9 (+ 0.21)	13.0 (− 0.26)	
6 (14 months)	Unrelated BM	FLU 25 mg/m2 × 5 L-PAM 90 mg/m2 × 2 rATG 1.25 mg/kg × 2	3.6 × 10^8^	Allele 7/8 match C 1 locus mismatch	FK506 short-term MTX	Day + 18	Day + 30	BM complete chimera at day + 33	no	No	No	4.2	105.0 (+ 1.14)	17.2(+ 0.85)	

*RIC* reduced-intensity conditioning, *HSCT* hematopoietic stem cell transplantation, *Pt* patient, *No* number, *HLA* human leukocyte antigen, *GVHD* graft-versus-host disease, *aGVHD* acute GVHD, *cGVHD* chronic GVHD, *SD* standard deviation, *CB* cord blood, *BM* bone marrow, *FLU* fludarabine, *L-PAM* l-phenylalanine mustard, *CY* cyclophosphamide, rATG rabbit anti-thymocytes globulin, *TLI* total lymphoid irradiation, *TAI* thoracic-abdominal irradiation, *TBI* total body irradiation, *CyA* cyclosporin A, *MTX* methotrexate, *FK506* tacrolimus, *MRSA* methicillin-resistant Staphylococcus aureus, *VOD* veno-occlusive disease, *GH* growth hormone

### Overall transplant outcomes: engraftment, complications and GVHD

**Fig. 2:**
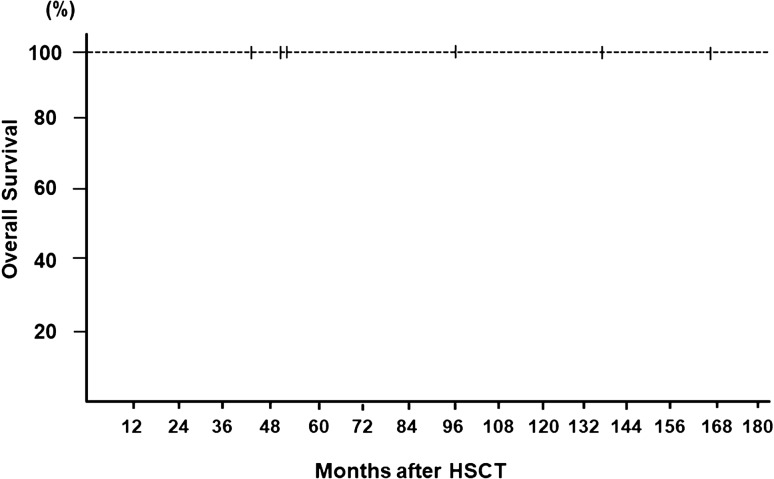
Overall survival rate after allogeneic hematopoietic stem cell transplantation in six patients with *MECOM*-associated syndrome

The overall survival rate after receiving HSCT was 100% (Fig. 2).

Neutrophil (Neut) and platelet (Plt) engraftments were successfully achieved in all patients between days + 6 and + 22, and between days + 22 and + 35, respectively. All patients achieved complete chimera of the donor type and independence from transfusion. No severe regimen-related toxicities were observed except grade 1 mucositis and veno-occlusive disease, which were treated with conventional therapies. Two patients presented with grade II acute GVHD of the skin that was easily controlled with 1 mg/kg prednisolone. None of the patients developed chronic GVHD.

### Hematological profiles and HSCT regimens of each patient

The hematological profiles and conditioning regimens for each patient are presented in Tables 1 and 2, respectively.

**Fig. 3:**
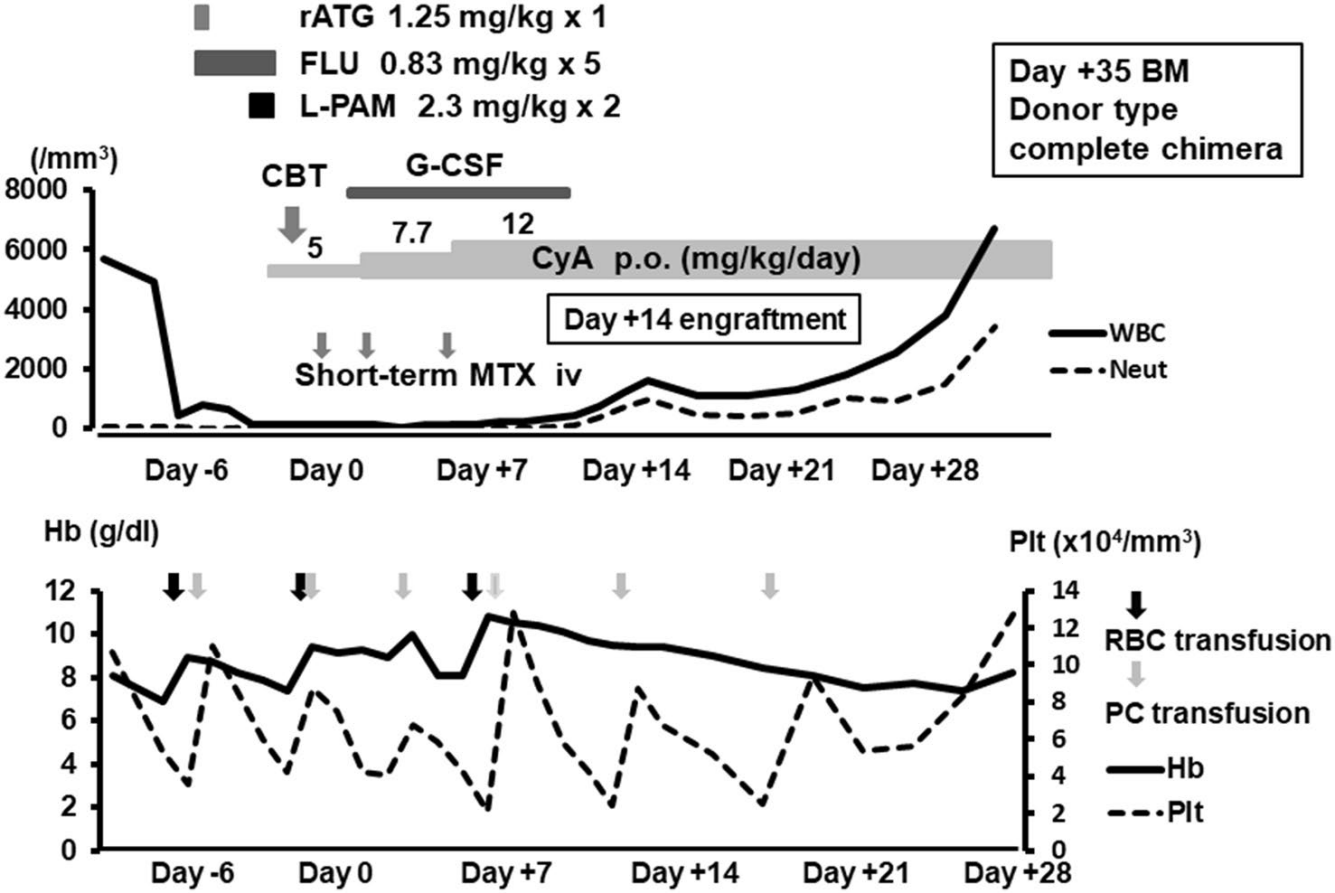
Clinical course of allogeneic cord blood transplantation in Pt 1

Pt 1 presented with severe fetal distress at birth (35 weeks and 6 days of gestation), and her birth weight was 2160 g. She appeared extremely pale due to severe anemia, and extensive petechiae were observed on her lower abdomen. Laboratory data revealed a normal white blood cell (WBC) count (6780/mm^3^) with neutropenia (Neut count 594/mm^3^), severe anemia (hemoglobin [Hb] level: 4.0 g/dL), and a low Plt count (5000/mm^3^). The patient required mechanical ventilation, red blood cell (RBC) transfusion, platelet concentrate (PC) transfusion, and treatment for hypovolemic shock. The patient’s general condition improved after treatment. Her bone marrow showed low cellularity without excess of blasts, absence of megakaryocytes or dysplasia. Radiographic images showed RUS of the bilateral forearms and bilateral bony defect of the intermediate phalanges of the fifth digits [4]. Neutropenia rapidly progressed, and PC transfusion was required twice a week. Hence, the patient immediately underwent allogeneic HSCT to prevent life-threatening infections at 4 months of age. Clinical course of allogeneic cord blood transplantation is shown in Fig. 3. Since there were no suitable conditioning regimens for CBT, fludarabine (FLU) (0.83 mg/kg for 5 days), melphalan (L-PAM) (2.3 mg/kg for 2 days) and rabbit anti-thymocyte globulin (rATG) (single dose of 1.25 mg/kg) were administered. HLA 1 allele-mismatched (DR) cord blood was selected, which contained enough total nuclear (21.2 × 10^7^/kg) and CD34 + (3.6 × 10^5^/kg) cells for engraftment. Oral cyclosporine A and short-term intravenous MTX were administered as GVHD prophylaxis due to limited blood access. After completing the conditioning regimen, the patient’s WBC count decreased to 0/mm^3^. She then achieved Neut and Plt engraftments on days + 14 and + 22, respectively. She developed grade 2 acute GVHD of the skin. However, the exanthema disappeared after administering 1 mg/kg of prednisolone. She achieved complete chimera of the donor type with sufficient recovery of megakaryocytes in the bone marrow on day + 35 and became independent from transfusion.

Pt 2 presented with massive systemic petechiae at birth (37 weeks of gestation), and her birth weight was 2058 g. Laboratory data revealed the following: 17,100/mm^3^ WBC count; 12.9 g/dL Hb level; and 8000/mm^3^ Plt count. Low Plt levels (< 10,000/mm^3^) persisted for 5 months and progressed to pancytopenia, requiring repeated RBC and PC transfusions. The patient’s radiographic image showed bilateral RUS and bilateral fifth digit clinodactyly, which caused limitations in forearm supination and pronation. Bone marrow examination revealed low cellularity and absence of megakaryocytes. She received allogeneic bone marrow transplantation (BMT) from an HLA full-matched donor with a sufficient total nuclear cells (6.9 × 10^8^/kg) at the age of 18 months. The conditioning regimen comprised FLU (25 mg/m^2^ for 4 days), cyclophosphamide (CY) (50 mg/kg for 4 days), rATG (2.5 mg/kg for 4 days), and total lymphoid irradiation (3 Gy). We administered FK506 and short-term MTX for GVHD prophylaxis. Neut and Plt engraftments were achieved on days + 16 and + 27, respectively. The patient presented with grade 2 acute GVHD of the skin, which was successfully treated with prednisolone [4, 8].

Pt 3 was born at 31 weeks of gestation, weighing 2180 g. The patient presented with severe pancytopenia at birth. Laboratory results showed 3220/mm^3^ WBC count, 48/mm^3^ Neut count, 2.7 g/dL Hb level, and 89,000/mm^3^ Plt count. Severe neutropenia (< 100/mm^3^) persisted and the patient’s platelet count decreased to < 20,000/mm^3^, requiring repeated RBC and PC transfusions. Moreover, antibiotics, antifungal agents, and immunoglobulin via intravenous infusion were administered to treat prolonged and repeated infections. The patient’s radiographic image showed bilateral RUS and overlapping fingers, which caused limitations in the forearm supination and pronation. Bone marrow examination revealed an absence of megakaryocytes. The patient received allogeneic BMT from an HLA 1 allele-mismatched (DR) donor with sufficient CD34 + cells (10.4 × 10^6^/kg) at the age of 8 months. The conditioning regimen comprised FLU (0.83 mg/kg for 5 days), CY (50 mg/kg for 4 days), and thoracic-abdominal irradiation (2 Gy). FK506 and short-term MTX were administered for GVHD prophylaxis. Neut and Plt engraftments were achieved on days + 6 and + 23, respectively. The patient only presented with grade 1 acute GVHD of the skin and was treated with a steroid ointment [4, 9].

Pt 4 was born at 40 weeks of gestation and had no remarkable family history of any illness. Her birth weight was 2936 g. She presented with massive pulmonary bleeding and developed respiratory insufficiency, requiring intubation and mechanical ventilation. Laboratory data revealed severe anemia (Hb level 7.2 g/dL) and thrombocytopenia (Plt count 9000/mm^3^) at birth. The patient presented with severe neutropenia that progressed to pancytopenia at 2 months of age. Bone marrow examination showed hypocellular marrow without megakaryocytes or dysplasia. Bone abnormalities were not observed. At the age of 5 months, we performed allogeneic CBT from HLA 1 allele-mismatched (DR1) cord blood due to recurrent life-threatening bacterial infection and transfusion dependency. The conditioning regimen consisted of FLU (1 mg/kg for 5 days), L-PAM (2.3 mg/kg for 2 days), and total body irradiation (3 Gy). FK506 and short-term MTX were administered as GVHD prophylaxis. Neut and Plt engraftments were achieved on days + 18 and + 35, respectively [6].

Pt 5 presented with systemic petechiae at birth (37 weeks of gestation), and her birth weight was 2368 g. She had severe bicytopenia (WBC count 10,700/mm^3^; Hb level 7.3 g/dL; and Plt count 4000/mm^3^). Hence, the patient required weekly PC transfusion since birth. Her father and uncle had RUS, and her brother, father, and uncle presented with clinodactyly of the fingers, but had no hematological abnormalities. Bone marrow examination revealed the absence of megakaryocytes, no excess of blasts and extremely low cellularity (3000/mm^3^). The patient presented with bilateral clinodactyly of the fifth digit. However, RUS was not observed. Emergent CBT was required due to transfusion dependency at the age of 8 months. The HLA compatibility of CB was DR 2 antigen mismatch (A/B/DR 4/6 match) and 4 allele mismatches (A/B/DR/C 4/8 match). The total and CD34 + cell counts at 17.21 × 10^7^/kg and 6.01 × 10^5^/kg, respectively, were sufficient for engraftment. The conditioning regimen in this patient was same as that of Pt 1. FK506 and short-term MTX were administered as GVHD prophylaxis. Neut and Plt engraftments were achieved on days + 22 and + 34, respectively. The patient only presented with grade 1 acute GVHD of the skin [7].

Pt 6 was born at 38 weeks and 6 days of gestation, and her birth weight was 3414 g. She had a maternal history of chronic thrombocytopenia (Plt count 41,000/mm^3^), transient leukopenia, bilateral RUS, congenital left clubfoot, and bilateral congenital disposition of the hip. The patient developed petechiae at birth, and laboratory data revealed severe anemia (Hb level 6.3 g/dL) and thrombocytopenia (Plt count 7000/mm^3^). No bone abnormalities were observed in the patient, and RBC and PC transfusions once per week were initially required. The transfusion dependency improved gradually, probably depending on somatic LOH in blood cells. Since Pt 6 remained PC transfusion-dependent, she was treated with allogeneic BMT from an HLA 1 allele-mismatched (C) unrelated donor at the age of 14 months. The conditioning regimen consisted of FLU (25 mg/m^2^ for 5 days), L-PAM (90 mg/m^2^ for 2 days), and rATG (1.25 mg/kg for 2 days). FK506 and short-term MTX were administered as GVHD prophylaxis. Neut and Plt engraftments were achieved on days + 18 and + 30, respectively. The patient did not present with any symptoms of acute GVHD, and all lineages of hematopoietic cells recovered well [7].

### Long-term sequelae after allogeneic HSCT

**Fig. 4:**
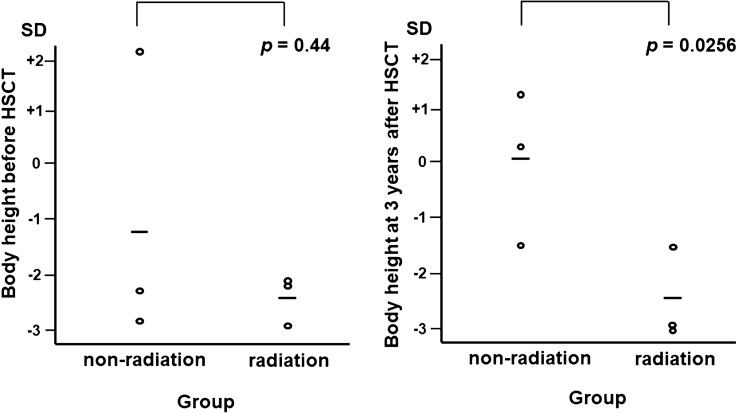
Risk of short stature in patients who received reduced-intensity conditioning regimens with low-dose irradiation. Standard deviations (SDs) of the mean body height before and 3 years after HSCT among patients who received low-dose irradiation (radiation group, *n* = 3) and those who did not (non-radiation group, *n* = 3) compared to age-matched healthy infants

All patients had good quality of life after allogeneic HSCT. However, there was no improvement in RUS nor in hearing disorders among the affected patients. In terms of long-term sequelae, we evaluated and compared the means and standard deviations (SDs) of body height between three patients who received irradiation (radiation group, *n* = 3) and three patients who did not receive irradiation (non-radiation group, *n* = 3) (Fig. 4). The body height in non-radiation group improved to normal levels of age-matched healthy infants after HSCT. However, the risk of short stature worsened at 3 years after HSCT in the radiation group. Pt 3 in the radiation group received growth hormone replacement therapy after HSCT. Nonetheless, this difference was not statistically significant due to limited number of patients. None of the patients have presented with secondary malignancies 3 years after RIC and allogenic HSCT.

## Discussion

RUSAT is a rare disease associated with IBMFS. The EVI1 protein plays an important role in maintaining normal hematopoiesis and hematopoietic stem cell functions. Hence, allogeneic HSCT is considered a reasonable curative treatment for *MECOM*-associated syndrome.

The heterogeneity of *MECOM*-associated syndrome has been a topic of interest because of the recent increase in the number of patients with *MECOM* mutations. In this case series, we assessed the broad clinical spectrum of *MECOM*-associated syndrome in six patients, including two patients with somatic mosaicism or LOH [6, 7]. In other current study, 6 of 179 children and young patients with undiagnosed IBMFS had *MECOM* mutations. None of the 6 patients had a remarkable family history, and four had no skeletal abnormalities. Moreover, only one had RUS [10].

All patients required allogeneic HSCT to overcome transfusion dependency and to prevent life-threatening infections in early infancy. However, there are two major problems with BMF treatment; the donor source for HSCT and the appropriate conditioning regimen. Regarding the donor source, a related or unrelated bone marrow donor can be chosen if HLA-matched donors are available. Appropriate cord blood is also applicable, as it can be urgently used compared to bone marrow from an unrelated donor in cases of emergent HSCT. Moreover, a sufficient number of infused cells is commonly available for infantile patients. In terms of conditioning regimens, they must be selected based on two conflicting issues, which are as follows: myelosuppressive effects for engraftment as well as lower incidence of regimen-related toxicities and long-term adverse effects, including short stature, endocrinopathy, infertility, and risk of secondary malignancy. Therefore, RIC regimens comprising FLU, alkylating agents, immunosuppressants, such as rATG and campath-1H, and/or low-dose irradiation have been used for nonmalignant diseases. rATG is an extremely strong immunosuppressant that eliminates T lymphocytes. Thus, it is not recommended as a conditioning regimen for CBT due to high mortality caused by delayed immune reconstitution, viral reactivation, and relapse of malignant diseases [11, 12]. The immunosuppressive effects of rATG are believed to be dose-dependent [13, 14], and thus, low-dose rATG was added to prevent long-term adverse effects caused by irradiation in Pts 1, 5, and 6. Three patients who received low-dose rATG for CBT did not show other adverse events such as delayed engraftment and viral reactivation. However, if the patient is at high risk of rejection owing to recipient T cell activation caused by viral infections or hemophagocytic syndrome, use of low-dose TBI and/or urgent second HSCT should be considered.

Long-term sequelae are critical in the management of infants who receive allogeneic HSCT. Irradiation at HSCT was found to be major factor for long-term height loss and relative risk for relevant growth deficiency increased in young patients [15]. Consistent with the previous report, patients treated with low-dose irradiation were at risk of short stature compared with patients without irradiation 3 years after HSCT in this study. However, the statistical significance and the difference among total body, thoracic-abdominal or total lymphoid irradiation remained undetermined due to limited number of patients in this case series. Of note, fatal cardiac complications during severe infections were reported in 2 of 6 patients after HSCT, which is a particular concern in patients with *MECOM*-associated syndrome [10]. The risk of malignancy in *MECOM*-related disorders has not been evaluated since the responsible gene was only identified in 2015 [4]. Approximately 44% of patients with familial platelet disorders that are predisposed to hematologic malignancies caused by autosomal dominant *RUNX1* mutations progressed to acute myeloid leukemia caused by second-hit mutations in *CDC25C* or other genes [16–18]. Moreover, alterations in EVI1 are involved in dysplastic hematopoiesis and acute leukemia of the megakaryocytic lineage in both humans and mice [19–23]. Therefore, patients with *MECOM* mutations may be at high risk of developing malignant diseases because of the long-term natural history of the disease or treatment with low-dose irradiation.

In conclusion, RIC regimens were feasible, and all infantile patients had perfect overall survival. In addition, they achieved stable, complete chimera of the donor type. Based on this retrospective study, we propose the RIC regimen comprised FLU, alkylating agents at appropriate doses, and low-dose rATG instead of low-dose irradiation if the patient is not at high risk of rejection to prevent the risks of short stature and secondary malignancy. Nevertheless, further investigations that include a larger number of infantile patients should be conducted to assess the optimal doses of alkylating agents and rATG in the RIC regimen followed by allogeneic HSCT in *MECOM*-associated syndrome.

## Data Availability

All data generated or analysed during this study are available on reasonable request.
